# “Trilongins” Offer Insight into Mold Toxicity

**DOI:** 10.1289/ehp.121-a44

**Published:** 2013-02-01

**Authors:** Bob Weinhold

**Affiliations:** Bob Weinhold, MA, has covered environmental health issues for numerous outlets since 1996. He is a member of the Society of Environmental Journalists.

Mold contamination inside buildings is a widespread occurrence that can cause a range of adverse health effects in certain people. But with tens of thousands of mold species and many mechanisms through which they may cause harm, much remains unknown about this realm of environmental exposures.^1^^,^^2^ Investigators now report that one mold, *Trichoderma longibrachiatum*, does some of its damage through a combined set of toxins previously unidentified for this species, at least two of which also had never been identified for any fungal species.^3^ The team of Finnish, Hungarian, and Russian researchers also determined that two classes of the toxin act synergistically to exacerbate the potency and duration of the toxic effects. They are unaware of any previous reports of such synergy.

Each mold species produces a unique set of toxins depending on species, strain, and growing conditions (e.g., temperature, incubation time, nutrient source, associated substances), with some uncertainty continuing to occur as toxin identification science is refined, according to principal investigator Mirja Salkinoja-Salonen, research director for the Department of Food and Environmental Sciences at Finland’s University of Helsinki. *T. longibrachiatum*, a fungus commonly found around the globe in a variety of damp indoor and outdoor settings, has been identified in a handful of case reports as an emerging human pathogen implicated in allergic sinusitis, lung and skin infections, and fatal postoperative infections in immunocompromised patients.

In the curren study the researchers began by exposing boar sperm cells to *T. longibrachiatrum* isolates taken from inside a Finnish moisture-damaged home, from the bodies of people with serious *T. longibrachiatum* infections, and from three diverse terrestrial locations (Wales, Egypt, and Antarctica). Exposure to *T. longibrachiatum* was linked to increased permeability, via voltage-dependent sodium and potassium ion channels, in some sperm cellular membranes. Additional observed damage, generally consistent with other research, included sperm motility inhibition and mitochondrial depolarization.

Normal ion channels perform vital cellular functions such as electrical and chemical signaling, regulation of cytoplasmic or vesicular ion concentration and pH, and cell volume regulation. They also maintain mitochondrial polarization, which is essential to the production of adenosine triphosphate (ATP), the molecule that acts as the medium of energy exchange for all cells. Dysfunctional ion channels are known to be key elements in a panoply of diseases, including epilepsy, cardiac arrhythmia, cystic fibrosis, kidney stones, hypertension, and various retinal, hearing, and skeletal-muscle diseases.^4^

**Figure:**
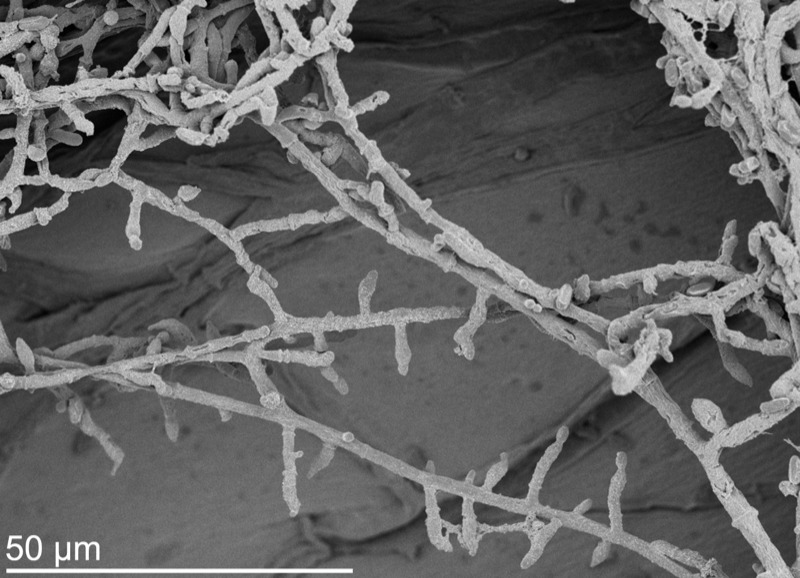
This sample of *T. longibrachiatum* was isolated from a moisture-damaged residence in Finland. After growing for 5 days at 37˚C, up to 2% of the fungal biomass by weight consisted of toxic trilongins. Maria A. Andersson, Mari Raulio, Mirja Salkinoja-Salonen

Further investigation by the team revealed that the substances involved in the ion channel damage contained various mixtures of two size classes of peptaibols—one 11-residue peptaibol and eight 20-residue peptaibols. Peptaibols are secondary metabolites formed by certain fungi that have been generally known for many years to alter ion channels in membranes.^5^

The team discovered that the peptaibols acted synergistically, with greater damage caused by isolates in which the two classes were combined. In toxicity tests, the half-maximal effective concentration of combined peptaibol classes was 5–25 times lower than that of single classes. Channels remained open about twice as long after exposure to combined classes, compared with exposure to a single class.

Salkinoja-Salonen says her team coined the term “trilongins” (“trilong-” is from “*Trichoderma longibrachiatum*,” and “-in” is the suffix for any peptaibol toxin) for the specific sets of peptaibols they identified to distinguish the unique group effect from that of single peptaibols. She says she and colleagues are working on additional studies investigating other fungal species, health end points, and toxins.

Boar sperm cells are good for studying mitochondria and mitochondrial toxins because the tail of the sperm contains many mitochondria that produce ATP, which is thought to power the flagellum, says Harriet Ammann, a toxicologist in Olympia, Wash-ing-ton, and a member of the committee that produced the 2004 U.S. Institute of Medicine publication *Damp Indoor Spaces and Health*.^6^ “Inhibition of motility is a sign of decreased or abolished mitochondrial function,” she explains.

Ammann points out that the documented mitochondrial effect could be critical since it indicates that a key function, energy production, could be significantly impaired. “Disabling of mitochondria could have widespread deleterious effects across systems, leading to greater susceptibility to infectious disease, an effect of this fungus already documented,” she says. In addition, she says, *Trichoderma* species produce other toxins with different mechanisms, which in all likelihood also work either additively or synergistically with peptaibols.

Identifying the ion channel damage pathway is important because there is little in the way of effective remedies for human trichodermal infections. “To my knowledge, treatment of [fungal-related] ion channel effects has not been developed,” Amman says. The only viable recourse for now, she adds, is to avoid exposure, saying that the new findings provide greater impetus to keep indoor environments dry and clean.
